# c-KIT expands the tool kit for treating arteriovenous malformation

**DOI:** 10.1172/JCI210364

**Published:** 2026-10-01

**Authors:** Ruilin Wu, Jason E. Fish

**Affiliations:** 1 Peter Munk Cardiac Centre, UHN Research Institutes, University Health Network, Toronto, Ontario, Canada.; 2 Department of Laboratory Medicine and Pathobiology, University of Toronto, Toronto, Ontario, Canada.

## Abstract

Hereditary hemorrhagic telangiectasia (HHT) is characterized by fragile, enlarged vessels with arteriovenous connections, which can form arteriovenous malformations (AVMs) that are prone to rupture. It is driven by mutations in TGF‑β pathway genes that disrupt endothelial cell responses and blood vessel remodeling. Two studies published in this issue of the *JCI* identify c‑KIT as a central mediator of HHT vascular pathology. Gahn et al. showed that SMAD4 loss derepressed c‑KIT, whose activity in the junctional mechanosensory receptor complex of endothelial cells reset the fluid shear stress set point to drive maladaptive vascular remodeling. Drapé et al. demonstrated that ALK1 loss in a mouse model of HHT induced a regional pro‑angiogenic c‑KIT^+^ endothelial state in brain AVMs. Both studies showed that pharmacologic c-KIT inhibition mitigated disease, revealing an unexpected, potentially ligand‑independent role for c‑KIT and highlighting a promising therapeutic target.

## Defective flow-mediated responses in HHT

Hereditary hemorrhagic telangiectasia (HHT) is a rare disease caused by mutations that dysregulate angiogenesis and vascular homeostasis. A hallmark of HHT is the presence of telangiectasias — fragile, distended vessels in the skin or mucosa that rupture easily and chronically bleed. These vessels have abnormal arteriovenous patterning, often bypassing the capillary bed through direct arteriovenous shunts (1). Arteriovenous malformations (AVMs) can also form in various organs, including the brain, where their rupture can result in hemorrhagic stroke. HHT is caused by mutations in TGF‑β pathway genes such as *ALK1*, ENDOGLIN (*ENG*), and *SMAD4*, leading to abnormal angiogenic and flow-mediated endothelial cell (EC) responses. Normally, increased blood flow promotes EC alignment, quiescence, and vessel remodeling through cell shape changes. In contrast, ECs lacking ENG are larger and show defective alignment in the direction of flow, resulting in vessel expansion (2). This is due in part to a lowered fluid shear stress (FSS) set point in HHT ECs, driving sustained activation of KLF4 and PI3K signaling and leading to continued maladaptive outward remodeling in an attempt to restore physiological wall shear stress levels (3).

## An unexpected role for c-KIT in flow sensing

The junctional mechanosensing signaling complex, which includes VE-Cadherin, VEGFR2, and PECAM1, plays a key role in EC responses to the physical forces of blood flow (4). Although expression of c-KIT (encoded by the *KIT* gene) is negligible in normal ECs, this receptor tyrosine kinase is upregulated in cells with reduced SMAD4 (5) or ALK1 (6) and is induced in ECs of mouse and human AVMs (5, 6). The activity of c-KIT in ECs has previously been implicated in regulating angiogenesis, including through modulation of glycolysis (7, 8). In a study published in this issue of the *JCI*, Gahn et al. unexpectedly discovered that although c-KIT has no impact on normal flow responses and is downregulated by laminar flow or BMP9 stimulation, it is stably incorporated into the mechanosensing complex in cells lacking SMAD4, where it plays a key role in lowering the FSS set point and augmenting flow signaling (5). Through mechanistic analyses, they found that c-KIT signaling acted in conjunction with the mechanosensing complex to sustain the MEKK3/ERK5/KLF4/AKT axis (Figure 1). Blocking c-KIT signaling restored cell shape and alignment with flow and reduced proliferation in *SMAD4*-knockdown ECs. This effect appeared to be independent of c-KIT’s main ligand, stem cell factor (SCF).

Several questions remain regarding the role of c-KIT in flow sensing. How does c-KIT signaling affect the activity of the other components of this complex? Since blocking c-KIT appears to suppress activation of Src family kinases, it may have a proximal role in flow responses that are upstream of the other components, but this remains to be established. Furthermore, while immunoprecipitation experiments demonstrate interaction between c-KIT and other mechanosensory proteins, the precise localization and regulation of the subcellular distribution of c-KIT requires further analysis. Why does c-KIT not have a role in flow responses under normal conditions? Is this simply due to reduced expression levels, or are there other aspects of signaling that are only activated in HHT ECs? Indeed, it is not clear what activates c-KIT signaling in the context of SMAD4 deficiency, since it appears to be SCF independent.

## Heterogeneity of HHT biology

In a related study in this issue, using regional analysis of brain AVMs (bAVMs), Drapé et al. discovered that EC-specific loss of *Alk1* instigated lumen expansion and AVMs in the perineural vascular plexus (PNVP) at the surface of the brain but not in the intraneural vascular plexus (INVP), suggesting that EC heterogeneity may influence pathogenesis (6). The loss of *Alk1* also led to the appearance of proliferative and proangiogenic c-KIT^+^ EC clusters specifically in the PNVP, which had heightened glycolysis and hypoxia signatures and resembled ECs from bAVMs in humans. Evidence of altered arteriovenous specification and blood-brain barrier signatures were also notable in these clusters. Trajectory analysis suggested that vein ECs are the site of origin of the c-KIT^+^ proangiogenic cluster. Interestingly, the PNVP is enriched in veins and has higher flow, while the INVP is mostly composed of capillaries with low flow; this may explain in part why lesions preferentially formed in this region. Additionally, the PNVP undergoes active hemodynamic remodeling during postnatal development, which may be a permissive environment for bAVM formation. Further work is needed to identify additional contributions of EC heterogeneity or elements of the microenvironment of the PNVP that are responsible for the susceptibility to AVM formation in this region. Additionally, future molecular studies are needed to integrate c-KIT with other known pathways driving HHT pathology (e.g., YAP/TAZ, RAS, Notch).

## Can c-KIT inhibition reverse established AVMs?

Since being a carrier of an HHT mutation brings with it a lifelong risk of developing AVMs, it is likely impractical to use c-KIT inhibitors as prophylactic interventions to prevent AVMs. Instead, treatment would likely begin once an AVM is detected. Establishing the extent to which targeting c-KIT may reverse pathology is therefore of paramount importance. Gahn et al. inhibited c-KIT signaling with avapritinib or masitinib in a postnatal retinal model of HHT (5). *Smad4* deletion was induced at postnatal day 0 (P0)/P1, c-KIT was inhibited at P3–P5, and lesions were analyzed at P6. This approach reduced the number of AVMs, the density of the vasculature, and proliferation markers and improved EC polarity. Interestingly, c-KIT inhibition at P4 did not reduce the number of AVMs, but it did reduce vessel diameter, suggesting that c-KIT targeting may have limited ability to reverse established lesions. Likewise, Drapé et al. used a drug-repurposing platform to identify c-KIT as an actionable target for HHT in a cerebrovascular postnatal model (6). They blocked c-KIT activity with masitinib, imatinib, or a c-KIT–blocking antibody at the time of *Alk1* deletion (P6) and analyzed bAVMs at P8 (right before lethality). Treatment reduced vascular area and vessel diameter. This outcome was accompanied by a suppression of the c-KIT^+^ angiogenic EC cluster and proliferation, but there was no change in the number or diameter of AV shunts, suggesting that c-KIT inhibition primarily affected EC proliferation but was not sufficient to reverse established shunts. Inhibition of c-KIT did not appear to affect normal vasculature, which is a favorable therapeutic advantage of targeting c-KIT.

As both groups utilized early postnatal developmental animal models, it will be of interest to investigate the activity and function of c-KIT signaling in advanced disease states. Future work should also utilize chronic models of HHT (e.g., ref. 9), since lesions form rapidly in the models used by Gahn et al. and Drapé et al., leading to lethality. Notably, c-KIT inhibition did not improve lethality in either model. These longer term models will be helpful for further assessing reversibility. Nevertheless, even if full reversion of AVMs is not possible, blocking progression could provide important clinical benefits, potentially including reduced bleeding from telangiectasias in the mucosa or reduced rupture of AVMs.

## What is the specificity of c-KIT inhibition?

The inhibitors used in the studies (i.e., avapritinib, masitinib, imatinib) are not completely specific to c-KIT signaling. For example, while masitinib has high activity against wild-type c-KIT, it can also inhibit PDGFR, Lyn, and FGFR3 and to a lesser extent ABL and c-FMS (10). Imatinib is the least specific of the three inhibitors, with strong inhibitory activity toward ABL and PDGFR, yet it is also the agent with the most clinical use, being well tolerated long-term in patients with leukemia (11). Additionally, c-KIT is known to be expressed in other cell types, such as macrophages and mast cells. While similar results across multiple c-KIT inhibition strategies is encouraging, genetic deletion of *Kit* specifically in the endothelium would be helpful to establish that c-KIT activity (and not other receptor tyrosine kinases) in the endothelium (and not other cell types) is responsible for phenotypic changes in the setting of HHT.

## Value of targeting c-KIT in other vascular anomalies

The findings of Gahn et al. and Drapé et al. identify c-KIT as an exciting potential target in HHT. It will be of interest to determine whether c-KIT may be involved in other vascular anomalies, such as sporadic AVMs and cerebral cavernous malformations (CCMs). Of note, *KIT* does not appear to be induced in ECs overexpressing mutant KRAS, the main genetic driver of sporadic bAVM (12). Drapé et al. showed limited c-KIT induction in one patient with sporadic brain AVM, but this will need to be replicated in a larger patient cohort with diverse causative mutations (6). While c-KIT signaling has not been examined in CCMs, the MEKK3/MEK5/ERK5/KLF2/4 signaling axis is well established to be involved in the pathogenesis of these lesions (13, 14). It is therefore reasonable to hypothesize that c-KIT signaling may be relevant to CCMs. Since c-KIT inhibitors would need to be used long-term in HHT, resistance mechanisms and toxicity will be important considerations. Future studies will reveal whether c-KIT signaling can be targeted to effectively and safely alleviate the dire consequences of vascular anomalies.

## Conflict of interest

The authors have declared that no conflict of interest exists.

## Funding support

This work is the result of NIH funding, in whole or in part, and is subject to the NIH Public Access Policy. Through acceptance of this federal funding, the NIH has been given a right to make the work publicly available in PubMed Central.

- JEF by the Canadian Institutes of Health Research (CIHR) PJT212866.
- JEF by the NIH 1R01HL159159.
- JEF by the UHN Canada Leads Program.
- RW from the following scholarships: Canadian Graduate Scholarship from CIHR, Ontario Graduate Scholarship, Queen Elizabeth II/Graduate Scholarships in Science and Technology from the Heart and Stroke Foundation, and the PRiME Next-Generation Precision Medicine Fellowship program from the University of Toronto.

## Figures and Tables

**Figure 1:**
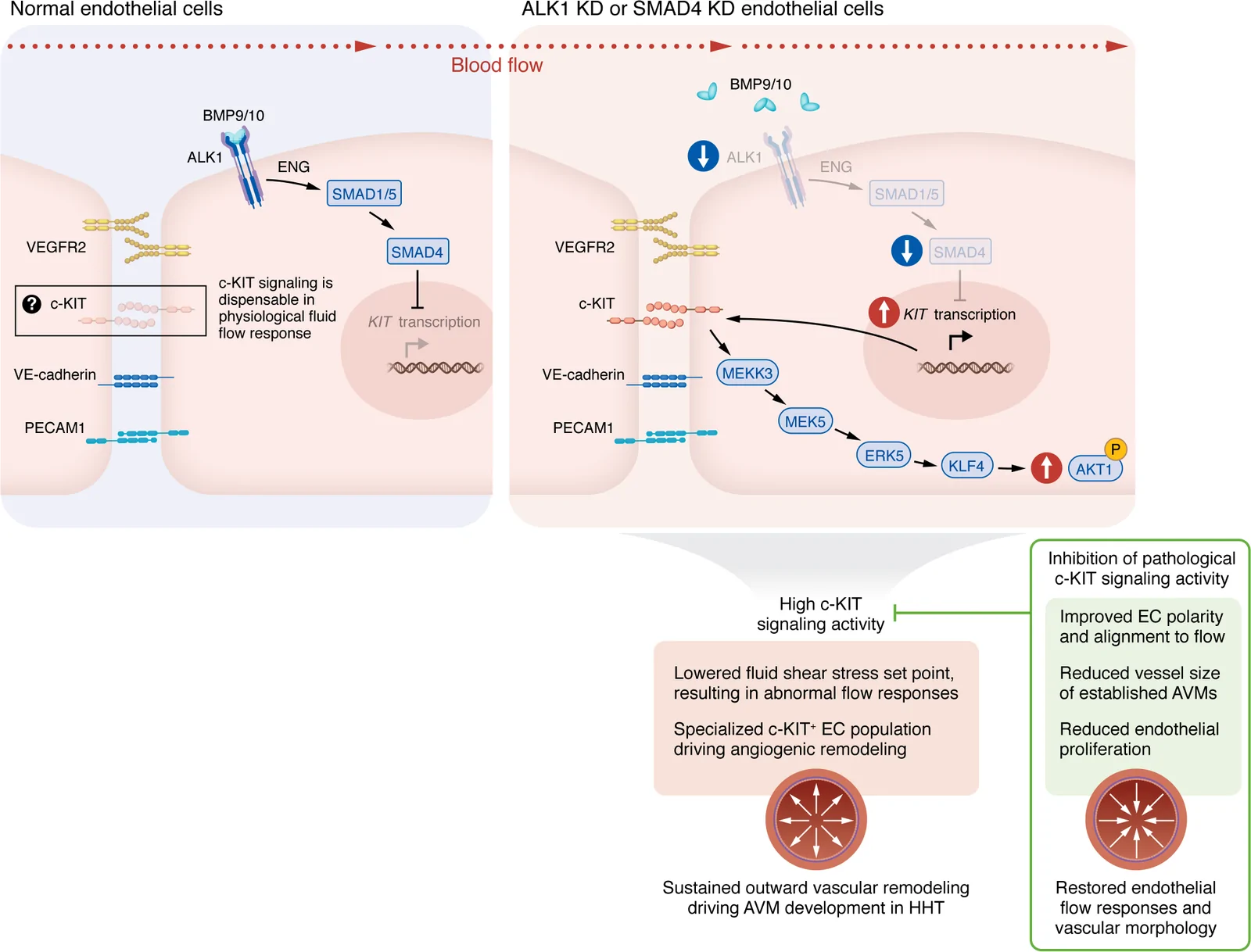
c-KIT as a mechanosensory component driving sustained outward remodeling and angiogenesis in HHT. Under physiological conditions, c-KIT expression in the endothelium is low, and c-KIT signaling is dispensable for blood flow responses and vascular development. However, related studies by Drapé et al. (6) and Gahn et al. (5) report that in the setting of HHT, loss of ALK1 or SMAD4 expression led to c-KIT upregulation and incorporation into the junctional mechanosensory complex. This resulted in excessive junctional signaling, leading to a lowered fluid shear stress set point in HHT ECs and sustained endothelial activation through the MEKK3/MEK5/ERK5/KLF4/AKT signaling axis. HHT ECs with high levels of c-KIT activity showed altered EC morphology and alignment, increased proliferation, and unique angiogenic signatures, driving continuous pathological vascular remodeling. Inhibition of c-KIT signaling in pathological ECs may restore these altered endothelial flow responses, offering a potential therapeutic direction for the management of AVMs in HHT.

## Version 1 10/01/2026

Electronic publication
